# Application of the New 2023 World Heart Federation Criteria to Rheumatic Heart Disease in the Setting of First Episode of Acute Rheumatic Fever: Predictors for Resolution

**DOI:** 10.1007/s00246-025-03966-7

**Published:** 2025-09-03

**Authors:** Jacqueline M. Williamson, Gillian A. Whalley, James Marangou, Peter S. Morris, Zhiqiang Wang, Joshua R. Francis, Bo Remenyi

**Affiliations:** 1 Menzies School of Health Research, 105 Rocklands Dr, Tiwi, NT 0810 Australia; 2 Charles Darwin University, Casuarina, NT Australia; 3 Department of Medicine, Otago School of Medicine, University of Otago, Dunedin, New Zealand; 4 Cardiology Department, Royal Perth Hospital, Perth, Australia; 5 Royal Darwin Hospital, Tiwi, NT Australia

**Keywords:** Rheumatic heart disease, Acute rheumatic fever, Mitral valve morphology

## Abstract

Rheumatic heart disease (RHD) is a chronic complication of acute rhematic fever (ARF). Echocardiography is used to assess valve disease in ARF. Acute valvulitis in ARF may normalise once inflammation has subsided. In the Top End of the Northern Territory (NT), a diagnosis of RHD is regularly made in conjunction with ARF diagnosis. We aimed to determine if baseline echocardiogram features could predict resolution of RHD when diagnosed during ARF. This retrospective cohort study includes children and young Australians diagnosed concurrently with first ARF and RHD between January 2012 and December 2021. Echocardiograms were reclassified based on the 2023 World Heart Federation guidelines for diagnosis of RHD. Primary outcome was echocardiographic resolution of RHD. The NT register recorded 311 individuals with concurrent diagnoses of ARF and RHD with 165 eligible for inclusion. Median age was 10 years (IQR 8–13 years), and 51.5% were female. Early RHD was diagnosed in 64% (106/165) of cases. Median follow-up time was 34 months. Resolution of RHD occurred in 19% (32/165) and 97% (31/32) of those had Early RHD at diagnosis. Absence of mitral valve leaflet thickening, restriction, and excessive leaflet motion were all associated with RHD resolution with univariate analysis. Multivariate Cox proportional modelling found that Early RHD at baseline independently predicted RHD resolution with HR of 16.6 (95% CI 2.25–122.74, *p* = 0.006). No difference was found between Stage A or Stage B RHD, (*p* = 0.461). The morphological features of valve disease were not as important as the severity in predicting resolution of RHD.

## Introduction

Rheumatic heart disease (RHD) is the chronic sequelae of acute rheumatic fever (ARF). This is due to an abnormal immune response to Group A Streptococcal (GAS) infection in susceptible hosts [1]. Acute rheumatic fever can result in (among other things) acute valvulitis, most commonly causing mitral and/or aortic regurgitation [2]. Following the period of acute inflammation, valvular lesions may persist, (or develop later). The characteristic morphological features of RHD and associated valve dysfunction are detailed in the World Heart Federation (WHF) guidelines for the echocardiographic diagnosis of RHD [3].

During the rheumatic process, the tips of the mitral valve leaflets may become inflamed and eventually fibrosed resulting in abnormal leaflet tip motion and leaflet restriction [4, 5]. The chords can also be affected with initial lengthening resulting in poor coaptation of the leaflets, followed by fibrosis and fusion causing chordal shortening which also results in poor leaflet coaptation and restriction. Commissural fusion causes mitral stenosis; however, this is less common in children [2]. This process also affects the aortic valve with inflammation of the cusps permitting prolapse and coaptation defect followed by thickening of the cusp edges, cusp restriction and, in older patients, stenosis [5]. These changes are captured in the WHF guidelines and form the basis of the criteria used to diagnose RHD once other causes of valve abnormality have been excluded [3].

The echocardiographic differentiation of chronic valvular changes associated with RHD and acute valvulitis accompanying the inflammatory response to GAS infection can be difficult, particularly if there is a history of previous ARF or RHD [6]. Restriction of leaflet motion often points towards chronic disease. Isolated valvular regurgitation, localised valvular thickening (beading) and excessive leaflet motion may be present in an acute or chronic setting [7, 8]. The WHF guidelines define pathological regurgitation but do not include details of the echocardiographic differentiation between acute and chronic morphological changes of the valves. This would be a useful addition to assist with prognostication at the time of ARF diagnosis, particularly given the high number of missed (or atypical) cases of ARF [9].

Approximately 50% of individuals with ARF experience carditis, and at least one quarter of those with ARF go on to have RHD [9]. One Brazilian study reported up to 72% of patients with ARF and carditis developed chronic RHD [10]. Data from the NT RHD register shows that many diagnoses of ARF and RHD are made concurrently in Australia [9]. This reflects reporting practices and suggests the presence of acute on chronic RHD in these individuals.

While we are familiar with the typical morphological features associated with chronic RHD, it is not known if there are particular echocardiographic features at diagnosis that predict normalisation of the valves following ARF. Others have reported on the clinical, biochemical and generalised echocardiographic characteristics associated with carditis regression [11, 12]. Our aim was to report on the individual valve features and associated pathology to determine which, if any, increased the likelihood of RHD resolution in young individuals diagnosed with ARF and RHD concurrently in an Australian population.

## Materials and Methods

### Population

All patients aged ≤ 20 years with first RHD diagnosis between January 2012 and December 2021 were identified from the Australian Northern Territory (NT) RHD register. For this retrospective cohort study, the subgroup of patients with concurrent diagnosis of apparent first ARF episode (within 3 months) were selected for inclusion. Acute rheumatic fever diagnoses were made using Australian guidelines [13] which are based on the Jones criteria [8, 14]. Cases of possible, probable and definite ARF diagnosed by clinicians are all regarded as ARF for the purpose of this study. Those with prior ARF, congenital heart lesions or absence of echocardiographic data at diagnosis or follow-up were excluded.

### Data Collection

Echocardiographic images from the time of RHD diagnosis (index echocardiogram) were reviewed by a single observer (JW, Observer 1). All studies were reported using the 2023 WHF echocardiographic guidelines for the diagnosis of RHD [3]. Results from Observer 1 were compared to the original diagnostic report generated by a single clinician (multiple reviewers, designated as Observer 2). In the event that valvular characteristics or lesion severity varied between observers, a third party (GW, Observer 3) reviewed the echocardiographic images to form a consensus decision. Observer 1 and Observer 3 were blind to the ARF status of each patient at the time of reviewing each echocardiogram. Observer 3 was also blind to the identity of each study and, therefore, the time interval between echocardiograms.

### Mitral Valve Morphology

**Fig. 1:**
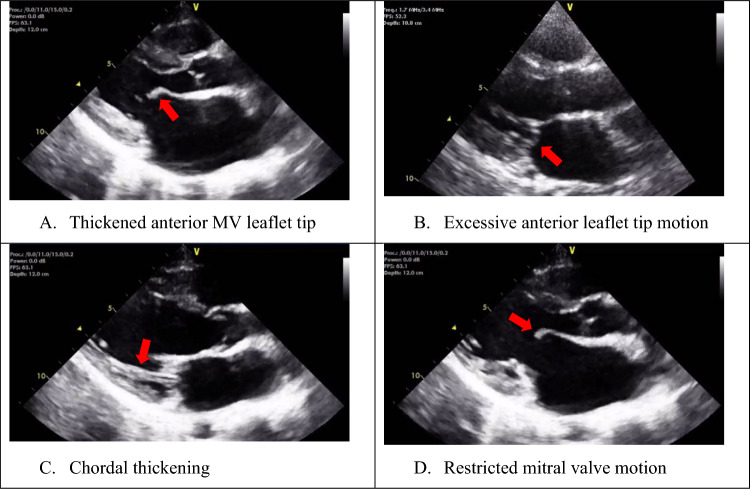
Mitral valve abnormalities associated with rheumatic heart disease. Parasternal long-axis views show **A** thickened anterior mitral valve leaflets (red arrow), **B** excessive anterior leaflet tip motion (red arrow), **C** chordal thickening (red arrow), and **D** restricted anterior leaflet tip motion (red arrow)

Four aspects of mitral valve morphology are assessed to diagnose RHD (Fig. 1).

### Aortic Valve Morphology

**Fig. 2:**
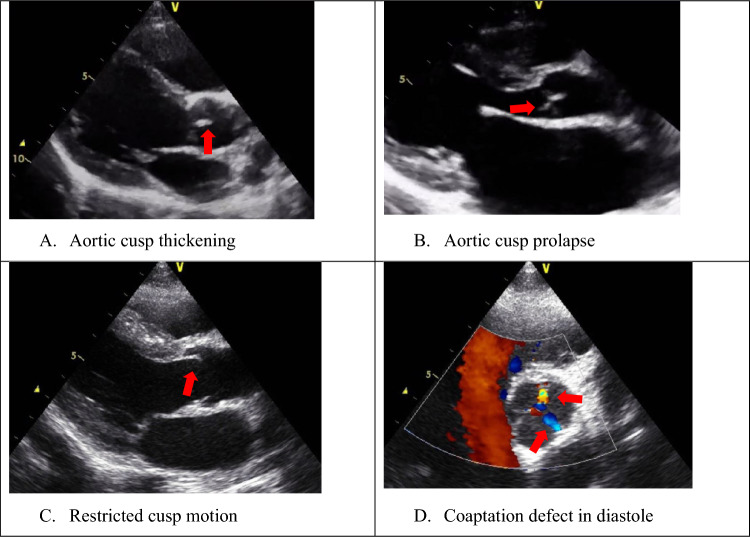
Aortic valve abnormalities associated with rheumatic heart disease. Parasternal long-axis views show **A** thickened aortic cusps (red arrows), **B** aortic cusp prolapse (red arrow), **C** restricted cusp motion (red arrow), and **D** parasternal short-axis view of the aortic valve in diastole demonstrating coaptation defect along the free edges of the valve with regurgitation seen in yellow and blue colour Doppler (red arrows)

Four abnormalities of the aortic valve are associated with diagnosis of RHD [3] (Fig. 2).

### Pathological Regurgitation

Pathological mitral regurgitation (MR) is defined by a jet that is: (i) seen in at least two views; (ii) ≥ 1.5 cm in length in those aged < 10 years, (otherwise ≥ 2 cm); (iii) ≥ 3 m/s throughout systole; and (iv) pan-systolic.

Pathological aortic regurgitation is defined by a jet that is: (i) seen in at least two views; (ii) ≥ 1 cm in length; (iii) ≥ 3 m/s in early diastole; and (iv) pan-diastolic.

All four Doppler criteria must be met to satisfy the diagnosis of pathological regurgitation of the relevant valve. These criteria are used to identify pathological valve regurgitation. Additional echocardiographic measures are needed to assess severity of disease [15, 16].

### Allocation of RHD Stage

Cases were classified as Stage A, B, C or D RHD based on the specific criteria detailed in the guidelines [3] (Table 1). Individual valve lesions including severity at index testing were recorded using the American Society of Echocardiography guidelines [15, 16].

**Table 1:** 2023 WHF guideline definitions of the rheumatic heart disease stages [17]

Stage A
Mild isolated pathological MR, or
Mild isolated pathological AR
Stage B
Mild pathological MR and mild pathological AR, or
Mild pathological MR with morphological abnormality/s^a^ of the mitral valve, or
Mild pathological AR with morphological abnormality/s^a^ of the aortic valve
Stage C
At least moderate MR, and/or
At least moderate AR, or
Any mitral stenosis, or
Any aortic stenosis
Stage D
Any of the Stage C lesions requiring surgical intervention^b^

^a^One or more morphological abnormalities required if ≤ 20 years, two or more abnormalities required if > 20 years

^b^The guidelines specify the presence of clinical complications including the need for surgical intervention constitutes Stage D RHD. In the Australian setting, surgery is an option for all those requiring it, and since this audit did not include clinical information beyond ARF and RHD diagnoses, surgical events provided by the RHD register were used to indicate the presence of Stage D RHD in this cohort

*AR* aortic regurgitation, *MR* mitral regurgitation, *WHF* World Heart Federation

All available echocardiograms prior to the index study were reviewed to rule out prior evidence of RHD. Echocardiograms subsequent to the index study were reviewed to determine if RHD had resolved. Follow-up time was determined by the number of months between index echocardiogram and either the date of first echocardiogram to demonstrate no RHD or the most recent echocardiogram in those that did not display resolution of RHD. This approach created the longest follow-up period of persistent valve disease possible.

Data regarding long-acting secondary antibiotic prophylaxis (SAP) dose dates and episodes of ARF were obtained from the NT RHD register. Time at risk of infection was calculated based on a 28-day regime of long-acting penicillin dose dates for each individual. The proportion of days at risk during the follow-up period was calculated by dividing the days at risk (from day 29 until the next dose date) by the total number of days of follow-up. The risk was expressed as a percentage of this time [13]. Antibiotic prophylaxis was considered adequate for those covered for ≥ 80% [13] of the follow-up period with results dichotomised as ‘adequate’ or ‘inadequate’ for regression analysis.

### Data Analysis

Results were captured in REDCap electronic data capture tool hosted at Menzies School of Health Research and transferred to Excel (Microsoft Corporation, 2018) for coding and preliminary analysis.

Statistical analysis was undertaken using Stata V14 (Stata Corp, Texas, USA). Continuous variables were reported as median and interquartile range (IQR) and categorical variables were reported as absolute number and percentage unless otherwise specified. Regression to No RHD was compared using Kaplan–Meier survival analysis. Log-rank testing was used to determine significance between stratified groups. Cox modelling was used for regression analysis with stepwise Akaike information criterion [18] used to determine the primary model. Sensitivity analysis was done to assess the impact of using alternative modelling strategies (including the removal of future ARF recurrences and SAP adherence from the analysis). A *p* value of 0.05 was considered statistically significant.

## Results

**Fig. 3:**
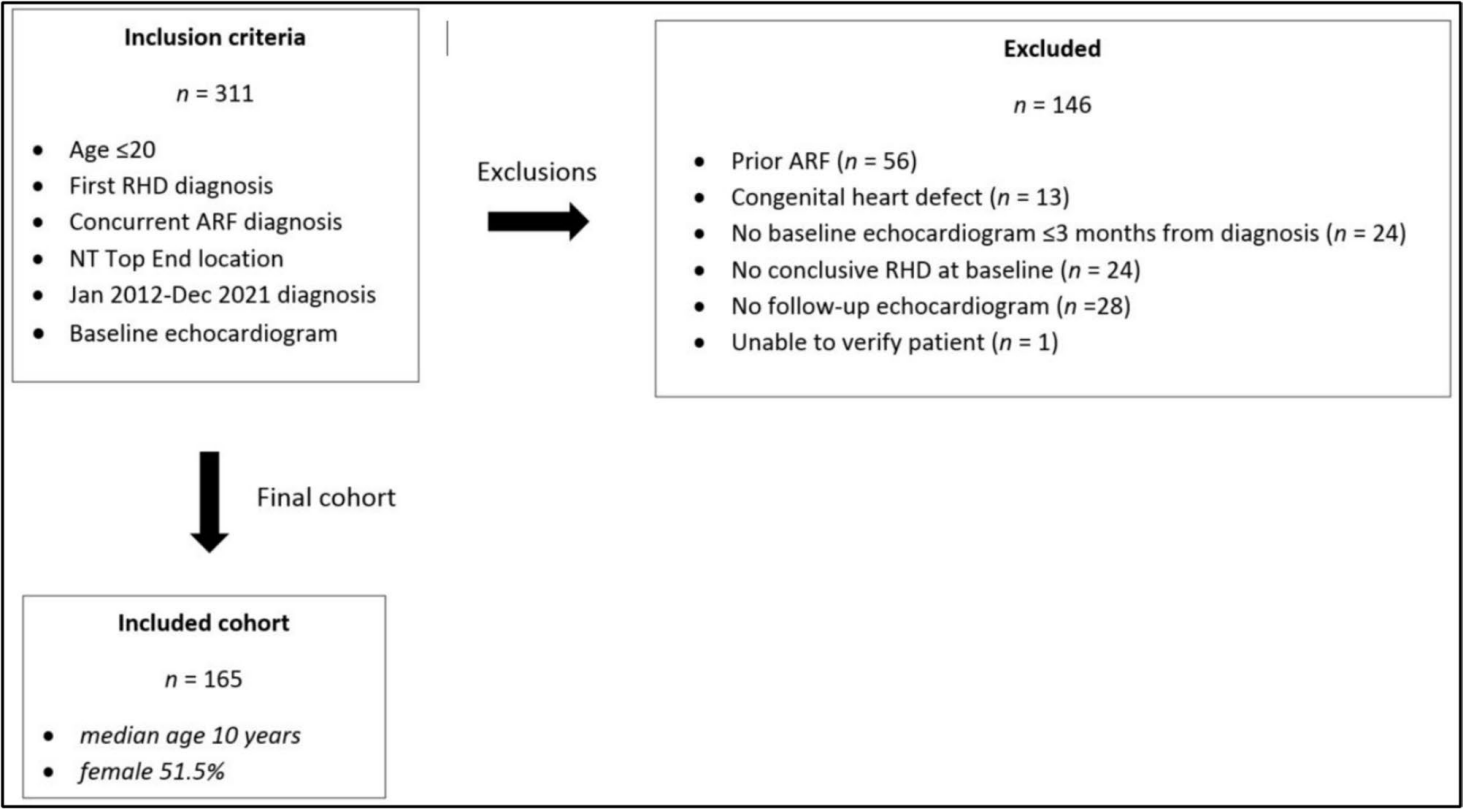
Derivation of sample population

Three hundred and eleven young individuals with concurrent RHD and ARF diagnoses were identified from the RHD register. Of these, 146 were excluded leaving a cohort (n) of 165 (Fig. 3). Sample population median age was 10 years, (IQR 8–13 years). Eighty-five (51.5%) were female and 99% (164/165) identified as Aboriginal Australian. Median length of follow-up for the group was 34 months (IQR 14–61 months). Third-party review was required in 52/165 cases (32%) due to either missing diagnostic reports or disagreement between Observer 1 and Observer 2.

Table 2 displays the baseline patient and echocardiographic characteristics for the group with resolved RHD (No RHD) and the group that retained echocardiographic features of RHD (chronic RHD) at follow-up beyond 6 months of the index echocardiogram. Univariate analysis demonstrated that Early RHD, isolated MR and absence of mitral valve leaflet thickening, excessive mitral valve leaflet tip motion and restricted mitral valve leaflet motion are all associated with resolution of RHD. Recurrence of ARF had a strong association with chronic RHD, (*p* = 0.005). No individual with ARF recurrence demonstrated resolution of RHD during follow-up.

**Table 2:** Patient demographics and baseline echocardiographic features

	No RHD at follow-up (*n* = 32)	Chronic RHD at follow-up (*n* = 133)	*p* value
Median age (IQR, years)	9 (7–12)	11 (8–13)	0.065*
Sex (female)	15 (46.9%)	70 (52.6%)	0.694
Median months of follow-up (IQR)	14 (11–19)	42 (19–70)	N/A
Number with adequate SAP (*n* = 136)	17 (63.0%)	61 (56.0%)	0.664
Median SAP adherence (IQR; *n* = 136)	84% (76–91%)	81% (64–90%)	0.127*
**Number with ARF recurrence**	**0 (0.0%)**	**24 (18.0%)**	**0.005**
**Baseline RHD stage**			** < 0.0001**
Early RHD—Stage A	15 (46.9%)	31 (23.3%)	
Early RHD—Stage B	16 (50%)	44 (33.1%)	
Advanced RHD—Stage C	1 (3.1%)	49 (36.8%)	
Advanced RHD—Stage D	0 (0%)	9 (6.8%)	
Baseline valvular characteristics			
**Pathological MR only**	**12 (37.5%)**	**25 (18.8%)**	**0.033**
Pathological AR only	3 (9.4%)	8 (6.0%)	0.450
**MV leaflet thickening**	**7 (21.9%)**	**72 (54.1%)**	**0.001**
MV chordal thickening	3 (9.4%)	34 (25.6%)	0.059
**MV excessive leaflet motion**	**1 (3.1%)**	**34 (25.5%)**	**0.003**
**MV restricted leaflet motion**	**7 (21.9%)**	**64 (48.1%)**	**0.009**
AV thickening	5 (15.6%)	32 (24.1%)	0.354
AV restriction	1 (3.1%)	15 (11.3%)	0.202
Coaptation defect	1 (3.1%)	8 (6.0%)	1.000
AV prolapse	0 (0.0%)	6 (4.5%)	0.598
**Normal valve morphology (MR ± AR only)**	**18 (56.3%)**	**39 (29.3%)**	**0.008**

*P* values are based on univariate comparisons between the group that resolved and the group with chronic RHD changes at follow-up with statistically significant results shown in bold

*t test, otherwise Fisher exact test (both two-sided)

*AV* aortic valve, *AR* aortic regurgitation, *ARF* acute rheumatic fever, *MR* mitral regurgitation, *MV* mitral valve, *RHD* rheumatic heart disease, *SAP* secondary antibiotic prophylaxis, *SD* standard deviation

### Severity (RHD Stage) of Disease at Index Testing

Stage D RHD individuals underwent early surgery and were excluded from time-to-event analysis (where resolution of RHD was the event of interest).

**Fig. 4:**
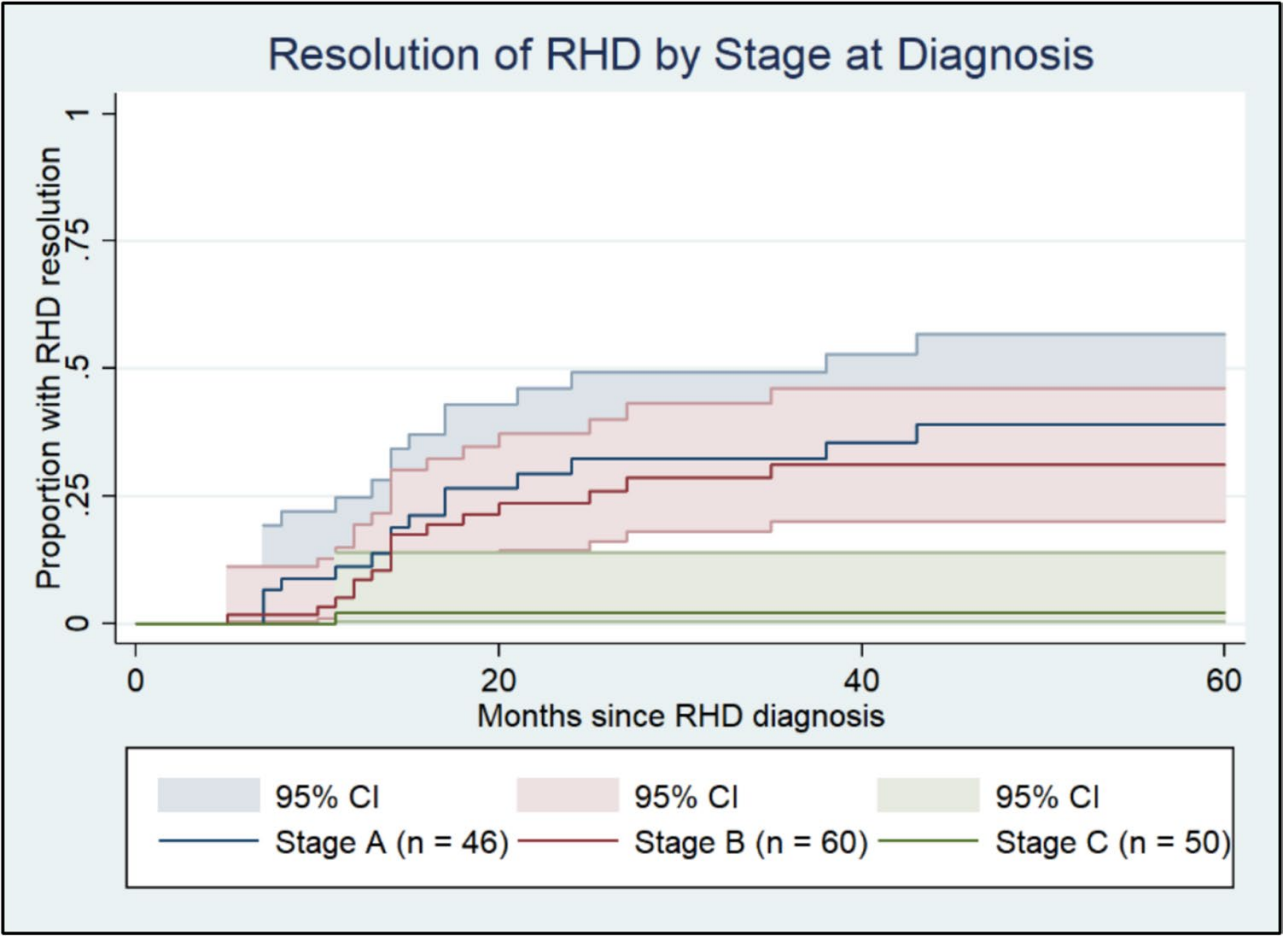
Kaplan–Meier curve demonstrates echocardiographic resolution of RHD in patients with concurrent ARF stratified by RHD stage at index testing. Patients with Early RHD at baseline (Stage A in blue, and Stage B in red) showed higher rates of resolution than those diagnosed with Stage C RHD (green)

Kaplan–Meier curve (Fig. 4) demonstrates superior outcomes for those with Early (Stage A and Stage B RHD). We found 11.2% (95% CI 4.80–24.8%) regression to no RHD at 1 year for Stage A; 8.6% (95% CI 3.7–19.5%) for Stage B; and 2.1% (95% CI 0.30–14.0%) for Stage C RHD (a single case). At 5 years, resolution of baseline changes occurred in 39.2% (95% CI 25.4–57.0%) for Stage A; and 31.4% (95% CI 20.3–46.5%) for Stage B. There was no further RHD resolution in Stage C at 5 years.

No difference was found between outcomes for Stage A and Stage B RHD (*p* = 0.461) (Fig. 4).

### Mitral Valve Morphology

The distribution of mitral valve leaflet thickening, chordal thickening and restricted leaflet motion was similar amongst those with Early and Advanced RHD (Table 3). Excessive leaflet motion occurred predominantly in those with advanced RHD. The presence of a single mitral valve morphological feature was infrequent, occurring in only 20% of those with abnormal morphology.

**Table 3:** Distribution of morphological mitral valve characteristics

	MV leaflet thickening (*n* = 79)	MV chordal thickening (*n* = 37)	MV excessive motion (*n* = 35)	MV restricted motion (*n* = 71)
Early RHD (Stage B)	41 (51.9%)	18 (48.6%)	7 (20.0%)	36 (50.7%)
Isolated feature	4 (5.1%)	3 (8.1%)	10 (28.6%)	4 (5.6%)
Isolated feature with Stage B RHD	4 (100%)	3 (100%)	2 (20.0%)	2 (50.0%)
Multiple features with Stage B RHD	37/75 (49.3%)	15/34 (44.1%)	5/25 (20.0%)	34/67 (50.7%)

*MV* mitral valve, *RHD* rheumatic heart disease

Excessive mitral valve leaflet motion was associated with resolution of RHD with hazard ratio (HR) of 8.86 (95% CI 1.19–65.87, *p* = 0.033, Table 4) when adjusted for other WHF defined structural and functional abnormalities of the mitral and aortic valves. Aortic valve abnormalities were combined into a single criterion for regression analysis due to low counts of individual features.

**Table 4:** Cox regression analysis comparing mitral and aortic valve abnormalities associated with diagnosis of RHD

No RHD at follow-up	Hazard ratio	95% CI	*p* value
No MV leaflet thickening	1.94	0.62–6.05	0.253
No MV chordal thickening	1.85	0.54–6.39	0.328
No MV restriction	1.60	0.51–4.97	0.417
**No MV excessive motion**	**8.86**	**1.19–65.87**	**0.033**
No AV morphological changes	0.93	0.35–2.47	0.888

Bold text shows statistically significant findings

*AV* aortic valve, *CI* confidence interval, *MV* mitral valve

**Fig. 5:**
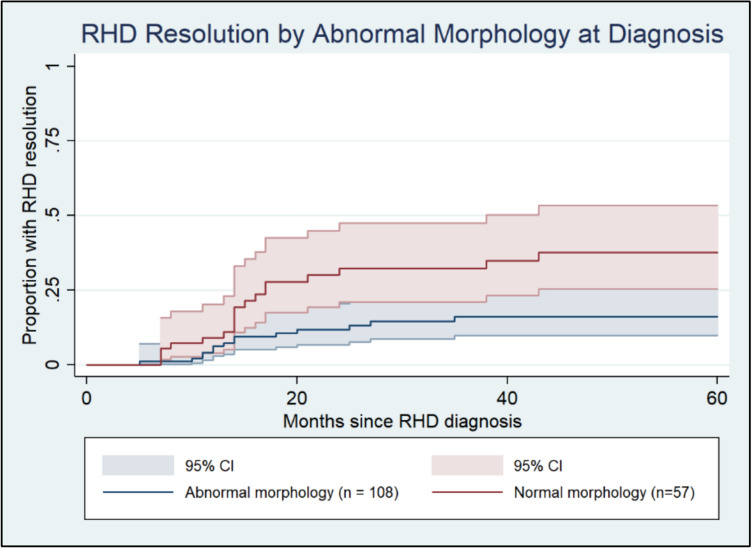
Kaplan–Meier curve demonstrating resolution of RHD stratified by the presence/absence of any (mitral or aortic) morphological valve changes

Univariate analysis demonstrated that individuals with normal morphology of the mitral and aortic valves were more likely to resolve with crude HR of 2.57 (95% CI 1.28–5.17, *p* = 0.008) (Fig. 5).

### Aortic Valve Morphology

No association was found between aortic valve morphological or functional changes and resolution of RHD.

### Multivariate Cox Regression Model

Covariates included for multivariate regression analysis were age, sex, SAP adherence (dichotomised as adequate or inadequate), ARF recurrence (dichotomised as yes or no), baseline RHD severity (dichotomised as Early or Advanced RHD), mitral valve leaflet thickening, mitral valve chordal thickening, excessive mitral valve leaflet motion, restricted mitral valve leaflet motion, aortic cusp thickening, restricted aortic cusp motion, aortic coaptation defect and aortic cusp prolapse. The final model after applying stepwise selection by Akaike information criterion is described in Table 5.

**Table 5:** Multivariate Cox regression showing predictors of RHD resolution

No RHD at follow-up	Hazard ratio	95% CI	*p* value
Age*	0.91	0.81–1.02	0.101
**Early RHD**	**16.77**	**2.27–123.74**	**0.006**
**No ARF recurrence**	**11.01**	**1.49–81.25**	**0.019**
No MV leaflet thickening	2.20	0.91–5.32	0.081
No MV chordal thickening	2.43	0.73–8.10	0.148

Stepwise selection by Akaike information criterion used to determine final regression model [18]. Statistically significant results shown in bold

*Age was analysed as a continuous variable

*ARF* acute rheumatic fever, *CI* confidence interval, *MV* mitral valve

Independent predictors of RHD resolution were Early RHD at baseline with HR of 16.8, (95% CI 2.3–123.7, *p* = 0.006) and absence of ARF recurrence with HR of 11.0 (95% CI 1.49–81.25, *p* = 0.019).

### Cox Regression Model Sensitivity Analysis with Inclusion of Alternative Variables and Exclusion of Future ARF Recurrences.

In a sensitivity analysis the mitral valve structural abnormalities of leaflet and/or chordal thickening were grouped, and the functional mitral valve abnormalities of restricted or excessive leaflet motion were grouped (as per the 2023 WHF guidelines). This model also adjusted for RHD severity and age but excluded ARF recurrence, because this risk factor is not available at the time of RHD diagnosis. No association was found between the morphological valve abnormalities and resolution of RHD (Table 6).

**Table 6:** Cox regression analysis evaluating the model after exclusion of future ARF recurrence and combination of mitral valve abnormalities as per the 2023 WHF guidelines

No RHD at follow-up	Hazard ratio	95% CI	*p* value
Age*	0.91	0.82–1.01	0.090
**Early RHD**	**13.81**	**1.78–106.98**	**0.012**
No MV thickening (leaflet/chord)	1.51	0.56–4.08	0.420
No MV restriction/excessive motion	1.48	0.52–4.19	0.459

Statistically significant results shown in bold

*Age was analysed as a continuous variable

*CI* confidence interval, *MV* mitral valve

### Normal Valve Morphology (Regurgitation Only)

A separate analysis considered the absence of all morphological valve abnormalities of both mitral and aortic valves together (no morphological valve abnormalities). This was not associated with resolution of RHD when adjusted for age, sex and baseline RHD severity (*p* = 0.547) (Table 7).

**Table 7:** Cox regression analysis evaluating a model after exclusion of ARF recurrence and absence of all morphological features

No RHD at follow-up	Hazard ratio	95% CI	*p* value
Age*	0.90	0.81–1.00	0.056
Sex	1.30	0.65–2.63	0.453
**Early RHD**	**17.30**	**2.28–131.12**	**0.006**
No morphological valve abnormalities	1.25	0.61–2.56	0.547

Statistically significant results shown in bold

*Age was analysed as a continuous variable

*CI* confidence interval, *RHD* rheumatic heart disease

### Alternative Multivariate Cox Regression Model

Cox regression model applying stepwise selection by Akaike information criterion was repeated after exclusion of future events of ARF recurrence and SAP adherence. Early RHD remained an independent predictor of resolution with HR of 16.96 (95% CI 2.25–122.74, *p* = 0.006). This was the only variable to independently predict RHD resolution in this model (Table 8).

**Table 8:** Multivariate Cox regression showing predictors of RHD resolution after excluding future events (ARF recurrence and SAP adherence)

No RHD at follow-up	Hazard ratio	95% CI	*p* value
Age*	0.92	0.83–1.03	0.151
**Early RHD**	**16.96**	**2.25–122.74**	**0.006**
No MV leaflet thickening	2.14	0.89–5.11	0.088

Stepwise selection by Akaike information criterion used to determine final regression model [18]. Statistically significant results shown in bold

*Age was analysed as a continuous variable

*CI* confidence interval, *RHD* rheumatic heart disease

## Discussion

Our data uniquely incorporate the 2023 WHF guidelines for the echocardiographic diagnosis of RHD [3] which we have retrospectively applied to acquired studies. These findings demonstrate that Early RHD (as opposed to Advanced RHD) is the most important independent predictor of RHD resolution when diagnosed in conjunction with ARF. This supports the definitions and associated risk of Early and Advanced RHD in the 2023 WHF guidelines [3] and aligns with previous Australian and international data demonstrating an association between RHD severity, disease progression and poor outcomes [11, 19–22].

Individual mitral valve abnormalities as defined by the WHF guidelines [3] were rarely found in isolation. Multiple morphological and structural changes involving leaflet thickening chordal thickening and restricted leaflet motion were found equally in those with Early and Advanced RHD. The presence of excessive mitral valve leaflet motion, whether in isolation or in combination with other features, was not a feature of Early RHD. This demonstrates that excessive leaflet motion is associated with higher grades of regurgitation (moderate or severe) that are classified as Advanced (Stage C or D) RHD.

The presence of aortic valve abnormalities has previously been linked to high risk of RHD progression [23]. Our cohort contained only a small number with aortic valve changes and we did not identify any association between aortic valve disease and RHD resolution.

We found no difference in RHD resolution between individuals with Stage A and Stage B RHD. Mild valvular regurgitation is present in both Early RHD stages. Stage A disease is defined by isolated mild mitral **or** mild aortic regurgitation. Stage B includes mild regurgitation of both valves or mild regurgitation with the addition of echocardiographically detected valve abnormalities. The WHF guidelines [3] provide different recommendations for those with Stage A or B RHD. Stage A RHD has a low risk of progression and cases may normalise after 1–2 years (and SAP ceased), while Stage B RHD is said to have a moderate–high risk of progression warranting longer periods of SAP. Our sample only included those with concurrent ARF who had echocardiographic imaging available at diagnosis and follow-up and may not represent the broader Early RHD population. Alternatively, it may be that the echocardiographic criteria defined in the WHF guidelines are unable to capture the underlying mechanism of mild pathological regurgitation in those with Stage A RHD. Mitral valve leaflet scallop separations have been reported in other populations as a cause for pathological (non-rheumatic) MR [24]; however, this study did not include evaluation of valve morphology beyond the criteria included in the WHF guidelines[3]. Larger prospective studies are required to better understand any differences in prognosis between Early RHD stages.

Almost 20% of individuals from this cohort demonstrated resolution of RHD with a median time to the detection of normalisation of 14 months after initial diagnosis. Some of these cases may represent acute valvulitis which resolved once inflammation subsided. The high proportion of Advanced RHD cases is consistent with a sample that is likely to include individuals with either: (i) undocumented episodes of prior ARF [25]; or (ii) an unusual clinical course that does not include previous episodes of typical ARF [26]. Recurrence of ARF is linked to disease progression [1]. Some of the children with apparent first episodes of ARF in this study may be cases of acute on chronic disease. Diagnostic overlap and limitations of echocardiographic evaluation make differentiation between acute valvulitis and chronic RHD with recurrent ARF difficult. Clinically, there may be limited benefit in separating the two conditions, since the management approaches are similar for both [13]. Differentiation may be important on an individual level, however. Telling a family that their child has chronic RHD has been associated with reduced quality of life [27, 28]. On the other hand, progression of Early RHD can be prevented with antibiotic prophylaxis, so early labelling of RHD may be beneficial in this population. Evolution of echocardiographic devices and the increase in artificial intelligent (AI) assistance [29–31] may improve the accuracy of RHD detection and permit improved delineation between acute carditis and chronic RHD.

## Limitations

Data regarding SAP was missing in 17% of patients including those prescribed oral antibiotics (because these data are not collected by the RHD Register). Our sample contained a small number of individuals with aortic valve changes. No association was found between aortic valve abnormality and regression to no RHD; however, this may represent a Type II error.

The valve characteristics detailed in the 2023 WHF guidelines [3] were used for analysis. The presence of mitral valve beading or nodularity associated with carditis during ARF [6] was reported as mitral valve or chordal thickening. Alternative approaches to categorising morphological changes in the valves could allow more accurate prediction of outcome.

This data set was obtained from young Aboriginal Australians living in the Northern Territory. Their clinical course may not represent the findings in other populations. Furthermore, evidence from this population may have been used to advise the 2023 WHF guidelines, introducing a small risk of incorporation bias.

## Conclusions

This retrospective cohort study sought to identify individual valve abnormalities associated with resolution of RHD diagnosed during ARF. Morphological abnormalities of the mitral and aortic valves were not able to predict resolution of RHD when adjusted for baseline severity. In our setting, only those with mild regurgitation were likely to resolve (whatever the morphological changes of their valves). Our results also reiterate established understanding of the poor prognosis associated with ARF recurrence and Advanced RHD at baseline, the absence of which both independently predicted RHD resolution.

Echocardiographic differentiation between acute valvulitis and chronic RHD with ARF recurrence is difficult due to overlap of diagnostic criteria. Further research is required to determine if the application of the 2023 WHF criteria to individuals with acute valvulitis is appropriate.

## Data Availability

Data can be made available upon request.

## Abbreviations

AV: Aortic valve
AR: Aortic regurgitation
AS: Aortic stenosis
ARF: Acute rheumatic fever
HR: Hazard ratio
IQR: Inter-quartile range
MR: Mitral regurgitation
MS: Mitral stenosis
MV: Mital valve
NT: Northern Territory of Australia
RHD: Rheumatic heart disease
SAP: Secondary antibiotic prophylaxis
WHF: World Heart Federation
